# Predicting benzodiazepine prescriptions: A proof-of-concept machine learning approach

**DOI:** 10.3389/fpsyt.2023.1087879

**Published:** 2023-03-10

**Authors:** Kerry L. Kinney, Yufeng Zheng, Matthew C. Morris, Julie A. Schumacher, Saurabh B. Bhardwaj, James K. Rowlett

**Affiliations:** ^1^Department of Psychiatry and Human Behavior, University of Mississippi Medical Center, Jackson, MS, United States; ^2^Center for Innovation and Discovery in Addictions, University of Mississippi Medical Center, Jackson, MS, United States; ^3^Department of Data Science, University of Mississippi Medical Center, Jackson, MS, United States

**Keywords:** benzodiazepine, prescriptions, machine learning, support vector machine, random forest

## Abstract

**Introduction:**

Benzodiazepines are the most commonly prescribed psychotropic medications, but they may place users at risk of serious adverse effects. Developing a method to predict benzodiazepine prescriptions could assist in prevention efforts.

**Methods:**

The present study applies machine learning methods to de-identified electronic health record data, in order to develop algorithms for predicting benzodiazepine prescription receipt (yes/no) and number of benzodiazepine prescriptions (0, 1, 2+) at a given encounter. Support-vector machine (SVM) and random forest (RF) approaches were applied to outpatient psychiatry, family medicine, and geriatric medicine data from a large academic medical center. The training sample comprised encounters taking place between January 2020 and December 2021 (*N* = 204,723 encounters); the testing sample comprised data from encounters taking place between January and March 2022 (*N* = 28,631 encounters). The following empirically-supported features were evaluated: anxiety and sleep disorders (primary anxiety diagnosis, any anxiety diagnosis, primary sleep diagnosis, any sleep diagnosis), demographic characteristics (age, gender, race), medications (opioid prescription, number of opioid prescriptions, antidepressant prescription, antipsychotic prescription), other clinical variables (mood disorder, psychotic disorder, neurocognitive disorder, prescriber specialty), and insurance status (any insurance, type of insurance). We took a step-wise approach to developing a prediction model, wherein Model 1 included only anxiety and sleep diagnoses, and each subsequent model included an additional group of features.

**Results:**

For predicting benzodiazepine prescription receipt (yes/no), all models showed good to excellent overall accuracy and area under the receiver operating characteristic curve (AUC) for both SVM (Accuracy = 0.868–0.883; AUC = 0.864–0.924) and RF (Accuracy = 0.860–0.887; AUC = 0.877–0.953). Overall accuracy was also high for predicting number of benzodiazepine prescriptions (0, 1, 2+) for both SVM (Accuracy = 0.861–0.877) and RF (Accuracy = 0.846–0.878).

**Discussion:**

Results suggest SVM and RF algorithms can accurately classify individuals who receive a benzodiazepine prescription and can separate patients by the number of benzodiazepine prescriptions received at a given encounter. If replicated, these predictive models could inform system-level interventions to reduce the public health burden of benzodiazepines.

## 1. Introduction

Benzodiazepines are the most commonly prescribed psychotropic medications in the U.S. (1), with approximately 12.5% of U.S. adults reporting past-year benzodiazepine use (2). They are known for their anxiolytic, sedative, hypnotic, relaxant, and anticonvulsant effects, and they are primarily indicated for short-term use in anxiety and sleep disorders (3, 4). Benzodiazepine use is associated with risk of serious adverse effects, such as psychomotor impairment, cognitive decline, falls, accidents, opioid overdose, substance use disorders, and death (5, 6), suggesting benzodiazepines pose a significant public health burden. Moreover, simultaneous receipt of multiple benzodiazepine prescriptions is considered a suboptimal and potentially high-risk prescribing pattern, as it can lead to increased plasma concentrations and risk of toxicity (7, 8), but there is a paucity of research on the correlates of multiple benzodiazepine prescription receipt. The present study aims to develop an algorithm to predict whether a patient is likely to receive a benzodiazepine prescription and the number of benzodiazepine prescriptions they are likely to receive at a given medical encounter, which could reduce the public health burden of benzodiazepine use and misuse by connecting patients with evidence-based treatments for anxiety or sleep disorders before they receive a prescription.

Research suggests access to benzodiazepines differs by demographic factors such as race, sex, and age. Indeed, multiple studies have found that in the U.S., White individuals are more likely than other racial groups to receive a benzodiazepine prescription (9, 10). Differences in the need for anxiety or insomnia treatment is unlikely to explain the variation in benzodiazepine prescriptions by race (11). Although the discrepant nature of benzodiazepine prescription rates by race may safeguard individuals from minoritized backgrounds from the risks associated with benzodiazepine use, they are also indicative of underlying disparities in screening for and treating anxiety and insomnia. Benzodiazepine rates have also been shown to differ by insurance status, such that individuals who are insured are more likely to receive a benzodiazepine prescription compared to patients without insurance coverage (12, 13). Another long-standing finding with benzodiazepine use is that women are more likely to use benzodiazepines than men (14), and female gender is associated with higher mean cumulative dosage of benzodiazepines (15). Moreover, male prescribers are more likely to prescribe benzodiazepines to female compared to male patients (15), which could indicate physician bias (e.g., male physicians may view their female patients as more anxious and in greater need of medication to treat their distress). Age is also associated with the likelihood of receiving a benzodiazepine prescription, with older patients more commonly receiving a benzodiazepine prescription (12, 16). Thus, a machine learning approach to identifying who is likely to receive a benzodiazepine prescription could not only help hospital systems begin to develop strategies to reduce the public health burden of benzodiazepines, but also identify disparities in the identification and treatment of anxiety and sleep disturbance by raising awareness of non-clinical factors that play a role in prescription prediction.

Additional research suggests individuals who are at the greatest risk of adverse benzodiazepine-related outcomes have an increased likelihood of receiving a benzodiazepine prescription (12). For example, patients with depression, schizophrenia, or a substance use disorder are prescribed benzodiazepines at higher rates than those without these conditions (12, 17–19). Similarly, individuals who are prescribed an antidepressant are more likely to be prescribed a benzodiazepine than those who do not use antidepressants (12). Individuals with a comorbid psychiatric or substance use disorder are at elevated risk of misusing benzodiazepines and of negative outcomes related to benzodiazepine use compared to the general population (17, 20, 21). Indeed, research suggests benzodiazepines are associated with new onset and worsening of depression symptoms (22) and that concurrent use of benzodiazepines with alcohol or opioids is associated with increased risk of emergency department visits, injury, overdose, and death (23–28). Furthermore, one study found that individuals with more severe chronic obstructive pulmonary disease (COPD) were more likely to receive multiple benzodiazepine prescriptions compared to those with less severe COPD (8). This finding is especially concerning given benzodiazepines’ respiratory depressant effect (29). As far as we are aware, no research has examined other clinical predictors of receipt of multiple benzodiazepine prescriptions. Developing an algorithm to predict who is likely to receive a benzodiazepine prescription and to stratify patients by the number of benzodiazepine prescriptions they are likely to receive at a given encounter represents an important first step toward reducing benzodiazepine prescriptions in these vulnerable populations.

To our knowledge, there is no predictive algorithm that exists to classify patients by their likelihood of receiving a benzodiazepine prescription or to stratify patients by the number of benzodiazepine prescriptions they are likely to receive at a given encounter. Machine learning uses computational modeling to learn from existing data, thereby improving predictive performance (30). The emergence of electronic medical records has led to the creation of large, rich sources of data that are ripe for health-related analyses which use machine learning to answer clinical questions more efficiently than traditional approaches (31). Specifically, machine learning methods can efficiently handle large numbers of predictors; capture complex, multidirectional, and non-linear relationships between variables; and classify clinically important populations (32). Prior research suggests machine learning can be used to predict patients’ risk for a variety of negative health outcomes (30, 31, 33), including sustained opioid prescription (34) or opioid overdose (35). Importantly, such an approach may help hospital systems begin to address issues of disparities in access to treatment for anxiety and sleep disorders and the use of potentially inappropriate prescriptions by raising awareness of non-clinical factors that are related to prescribing. The current study aims to apply machine learning methods to develop algorithms for stratifying patients by the likelihood of receiving a benzodiazepine prescription and the number of benzodiazepine prescriptions they are likely to receive at a given encounter.

## 2. Materials and methods

### 2.1. Data source

**FIGURE 1:**
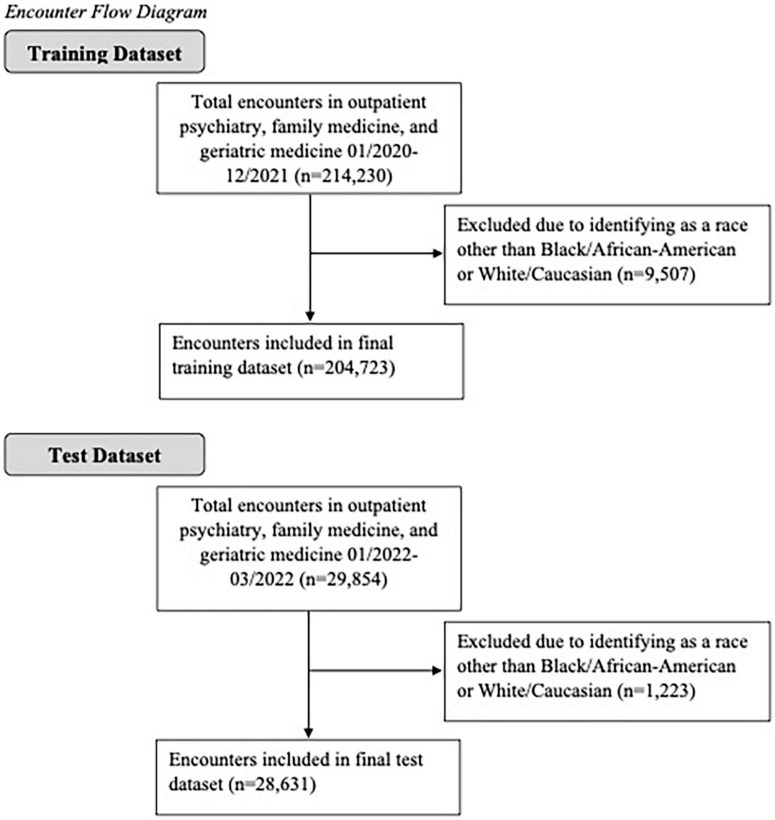
Encounter flow diagram.

Electronic health record data were obtained from a research data warehouse at an academic medical center (36). Data for this study were de-identified and date-shifted and thus did not include any protected health information. The data warehouse compiles data from the electronic records system, Epic, based on encounters at all of the institution’s hospitals and clinics. The present study included data from encounters in three specialties: family medicine, outpatient psychiatry, and geriatric medicine, as benzodiazepines are most commonly prescribed in these settings (14). All patients who identified their race as either Black/African-American or White/Caucasian were included; all other races were excluded, as only 4.2% of encounters in the training dataset and 3.6% of encounters in the test dataset were with patients who identified as another race (see Figure 1 for a diagram of included encounters). This proof-of-concept study builds on prior research by using machine learning to identify demographic and clinical factors to improve prediction of benzodiazepine use in patients seen at Mississippi’s only academic medical center.

### 2.2. Features and outcomes

The following sets of features were selected: anxiety and sleep diagnoses (four features: primary anxiety disorder diagnosis, any anxiety disorder diagnosis, primary sleep disorder diagnosis, any sleep disorder diagnosis), demographic characteristics (three features: age, gender, race), medications (four features: opioid prescription at encounter, number of opioid prescriptions at encounter, antidepressant prescription at encounter, antipsychotic prescription at encounter), other clinical variables (four features: any mood disorder diagnosis, any psychotic disorder diagnosis, any neurocognitive disorder diagnosis, prescriber specialty), and insurance status (two features: any insurance, type of insurance). Breathing-related sleep disorder diagnoses were excluded because benzodiazepines are contraindicated for these disorders (6). The outcomes of interest were whether a benzodiazepine was prescribed at the encounter (yes/no) and the number of benzodiazepine prescriptions given to the patient at the encounter (0, 1, 2+). Benzodiazepine prescriptions included: alprazolam, clonazepam, diazepam, chlordiazepoxide, clorazepate, lorazepam, midazolam, and temazepam.

### 2.3. Analytic approach

We used the Statistical Package for Social Sciences (SPSS, version 28.0) for data management and MATLAB R2020b for analysis. We used multiple machine learning approaches to determine how to optimize prediction of benzodiazepine prescriptions. Specifically, we applied support vector machine (SVM) and random forest approaches to the de-identified electronic health record data for encounters in psychiatry, family medicine, and geriatrics at an academic medical center. To train and test the prediction models, two separate datasets were collected. The training dataset was collected between January 2020 and December 2021, while the test dataset was collected between January 2022 and March 2022. Overall patient-level sample characteristics are presented for the training and test datasets in Supplementary Table 1. No cross validations (e.g., k-fold) were applied to our analyses, as the test dataset was completely separated from the training dataset. All prediction results reported in the following tables and figures were derived from the test dataset. To compare the performance of different algorithms, the true positive rate (i.e., the ratio of values that are predicted to be positive and are actually positive to all positive values), the true negative rate (i.e., the ratio of values that are predicted to be negative and are actually negative to all negative values), and the overall accuracy were calculated. Some research suggests the area under the receiver operating characteristic curve (AUC) is a better measure for evaluating the predictive ability of machine learning algorithms compared to accuracy (37); therefore the AUC was also calculated for the models predicting benzodiazepine receipt. The AUC was not calculated for models predicting the number of prescriptions, as receiver operating characteristic curves are not suitable for multi-class classifications. The AUC is a function of both sensitivity and specificity and can be interpreted such that a value of 1.0 is a perfect test of classification, 0.90–0.99 is considered excellent, 0.80–0.89 is considered good, 0.70–0.79 is considered fair, 0.51–0.69 is a poor test, and a value of 0.5 corresponds with no improvement in prediction over chance (38, 39). A total of 17 features were selected *a priori* based on existing literature. Given the small number of features and the large sample size, we opted to manually combine different sets of features to test the classification accuracy, which can be more easily interpreted than using data-driven approaches to feature selection. A model building approach was used to determine which sets of features would maximize predictive accuracy. The models tested were as follows:

Model 1: Anxiety and Sleep Diagnoses Only.

Model 2: Anxiety and Sleep Diagnoses + Demographic Characteristics.

Model 3: Anxiety and Sleep Diagnoses + Demographic Characteristics + Co-Prescriptions.

Model 4: Anxiety and Sleep Diagnoses + Demographic Characteristics + Co-Prescriptions + Other Clinical Variables.

Model 5: Anxiety and Sleep Diagnoses + Demographic Characteristics + Co-Prescriptions + Other Clinical Variables + Insurance.

All training samples were used for training. Undersampling was employed on the training dataset to avoid bias given the unequal distribution of negative responses (i.e., did not receive a benzodiazepine prescription) compared to positive responses (i.e., received a benzodiazepine prescription). Suppose there are *m* samples of benzodiazepine prescription (yes), and *n* (typically *n* > *m*) samples of non-benzodiazepine prescription (no). We randomly selected *n*′ (= *m*) samples from *n* samples. Each model was repeated 30 times, yielding 30 different *n*′ samples. A multivariate analysis of variance (MANOVA) was performed for each model to compare whether mean performance differed between the random forest and SVM approaches. The SVM and random forest algorithms were implemented on the Matlab R2020b platform using default settings, except where noted otherwise.

#### 2.3.1. Support vector machine

Support vector machine is a supervised learning model that analyzes data and performs non-linear classification (40). When provided a set of training data, in which each observation is coded as belonging to a group, an SVM training algorithm uses the data to build a model that can assign new data points to a specific category. An SVM creates a hyperplane (or set of hyperplanes), or a separating line between data belonging to different classes, for classification. It seeks to identify the optimal hyperplane by maximizing the distance between the hyperplane and the closest data points in each class. By maximizing the distance between the hyperplane and the nearest data points in each class, the SVM model minimizes the generalization error of the classifier (41).

For the present analyses, in the SVM method, we used a Gaussian kernel function and a one-versus-one coding design, which yields two (or three) binary learners and for two (or three) classes. To create a receiver operating characteristic curve, we transformed SVM classification scores to class posterior probabilities, which are obtained by predicting the maximum class posterior probability at each point in a grid.

#### 2.3.2. Random forest

*Random forest* is another supervised learning model that can be used for classification. It uses ensemble learning, meaning it combines multiple models to solve complex problems, rather than using an individual model (32). The random forest algorithm relies on bagging or bootstrap aggregating to improve accuracy. It uses random subsets of a training dataset to generate individual decision trees for each subsample. Each decision tree will produce an output (i.e., a classification). The final output is chosen based on “majority voting;” in other words, the random forest output is the class that is chosen by the most trees. The random forest approach can reduce the effects of overfitting in individual decision trees (42).

For the present study, in the random forest model, we trained an ensemble of 100 classification trees using the entire training dataset. A random subset of predictors was used at each decision split. The selection of the split predictors aims to maximize the split-criterion gain over all possible splits of all predictors. The number of candidate predictors considered for each tree (i.e., mtry) differed for each model such that $m\,t\,r\,y=r\,o\,u\,n\,d\,u\,p\,\left(\sqrt{\#\,F\,e\,a\,t\,u\,r\,e\,s}\right)$. Random subsets of the training dataset were sampled with replacement. The final classifications are the combined results of all trees.

#### 2.3.3. Feature selection

It should be noted that a model with few predictors is preferred, as it is less costly and time-consuming to use (43). To address this concern, many choose to employ data-driven feature selection approaches, e.g., (44) to remove non-informative features from models. Methods for data-driven feature selection include wrapper methods, which evaluate multiple models by adding and/or removing features to optimize model performance, and filter methods, which assess the relevance of features separately from the predictive models and only include predictors that meet specified criteria in the final model (43). However, both approaches have disadvantages. Wrapper methods involve the evaluation of many models, which significantly increases computation time, and it can increase the risk of over-fitting the model (43). In contrast, filter methods are more computationally efficient, but they involve using selection criteria that are not necessarily related to the optimization of the model. Moreover, because each feature is evaluated separately, it is possible that redundant features are selected for the final model, while interactions between features are not quantified during the feature selection process (43).

In addition, tree-based algorithms, such as random forest, conduct feature selection automatically. For instance, during the construction of a tree, if a feature is not employed in any split, the model is effectively independent of the feature (43). In fact, prior research suggests tuning random forest models can reduce the effect of non-informative features (45), precluding the need for feature selection in random forest approaches. Conversely, random forest is a powerful classifier because it can utilize weak features, which may be suppressed by methods such as principal component analysis, to boost the classification performance.

In the present study, we used relatively few features (i.e., 17 features), which were selected *a priori* based on existing literature. We have previously employed this approach (46), resulting in improved accuracy when compared to data-driven feature selection. Given the small number of features and the large sample size, we opted to manually combine different sets of features to test the classification accuracy, which can be more easily interpreted than using data-driven approaches to feature selection.

## 3. Results

In the training dataset, collected between January 2020 and December 2021, there were a total of 204,723 encounters taking place at outpatient psychiatry, family medicine, or geriatric medicine (involving 37,979 patients); there were 4,424 encounters at which a patient received at least one benzodiazepine prescription, while there were 200,299 encounters where a patient received no such prescription. Of these, there were 3,988 encounters where a patient received one benzodiazepine prescription and 436 encounters where a patient received two or more benzodiazepine prescriptions. Patient-level characteristics for the training dataset are presented by benzodiazepine prescription status in Supplementary Table 2.

In the test dataset, collected between January 2022 and March 2022, there were a total of 28,631 encounters (involving 14,404 patients); there were 842 encounters at which a patient received at least one benzodiazepine prescription and 27,789 where a patient received no such prescription. In the test data, there were 792 encounters where a patient received one benzodiazepine prescription and 50 encounters where a patient received two or more benzodiazepine prescriptions. Because the number of “positive” observations (i.e., received a benzodiazepine prescription) is significantly lower than the number of “negative” (i.e., did not receive a benzodiazepine prescription) observations, the number of positive and negative observations were balanced prior to model training in order to avoid bias. Patient-level characteristics for the test dataset are presented by benzodiazepine prescription status in Supplementary Table 3.

All prediction results (e.g., accuracy, AUC) reported in the following tables and figures were derived from the test dataset.

### 3.1. Prescription receipt

**FIGURE 2:**
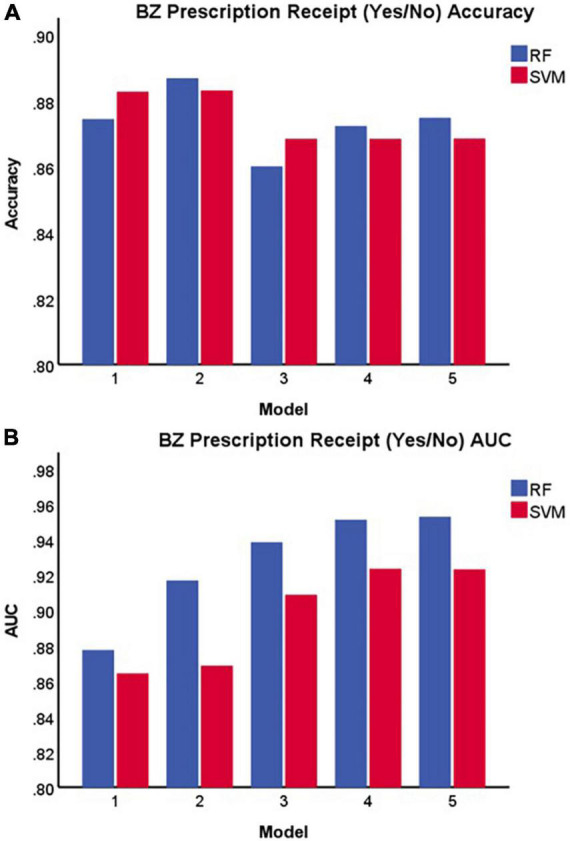
Accuracy **(A)** and area under the receiver operating characteristic curve **(B)** of each model in predicting benzodiazepine prescription receipt at a given encounter (yes/no). BZ, benzodiazepine; SVM, support vector machine; RF, random forest; AUC, area under the receiver operating characteristic curve.

**FIGURE 3:**
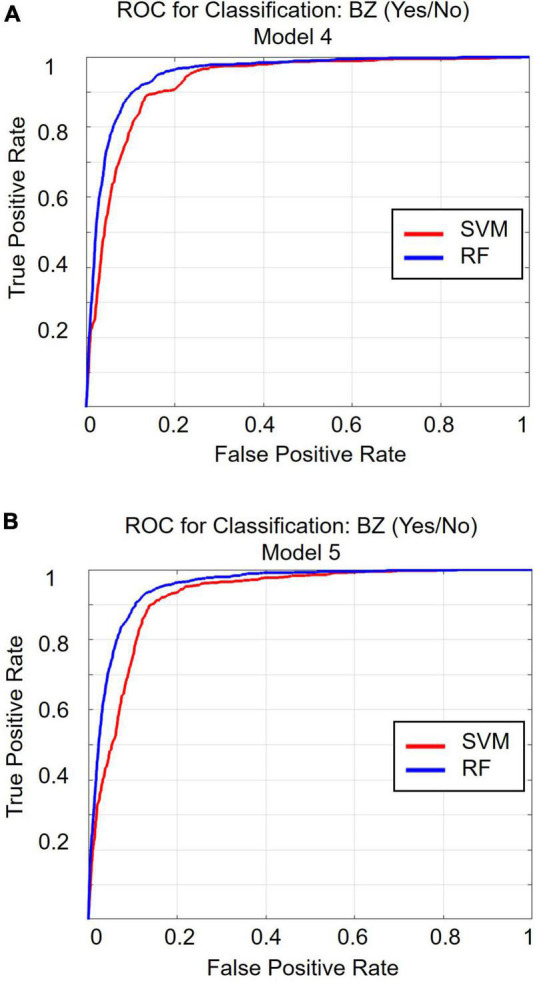
Receiver operating characteristic curve for classification Model 4 **(A)** and for classification Model 5 **(B)**. ROC, receiver operating characteristic curve; BZ, benzodiazepine; SVM, support vector machine; RF, random forest.

Table 1 and Figure 2 display the results for the models predicting whether a patient received a benzodiazepine prescription at a given encounter (yes/no). As depicted in Figure 2A, for the SVM approach, overall accuracy did not improve after including the first set of features (i.e., anxiety and sleep diagnoses). For the random forest approach, Model 2 maximized overall accuracy when predicting whether a patient received a benzodiazepine prescription at a given encounter (yes/no), and the random forest model slightly outperformed the SVM model (Random Forest benzodiazepine prescription receipt Model 2 accuracy = 0.887; SVM benzodiazepine prescription receipt Model 1 accuracy = 0.883, *F*(1, 58) = 52.892, *p* < 0.001). However, as shown in Figure 2B and Figure 3, when examining the AUC, Model 4 maximized the AUC for the SVM approach when predicting benzodiazepine prescription receipt at an encounter (SVM benzodiazepine prescription receipt Model 4 AUC = 0.924), while Model 5 maximized the AUC for the random forest approach when predicting whether a patient received a benzodiazepine prescription (Random Forest benzodiazepine prescription receipt Model 5 AUC = 0.953).

**TABLE 1:** Benzodiazepine prescription receipt (yes/no) prediction results.

		Support vector machine		Random forest		Comparison	
	Number of features	M (SD)	95% CI	M (SD)	95% CI	*F*	*p*
Model 1	4					37.940	<0.001
Accuracy		0.883 (0.003)	0.882–0.884	0.874 (0.005)	0.873–0.876	58.935	<0.001
TPR		0.828 (0.008)	0.824–0.831	0.850 (0.014)	0.845–0.855	58.935	<0.001
TNR		0.884 (0.003)	0.883–0.886	0.875 (0.006)	0.873–0.877	58.935	<0.001
AUC		0.864 (0.016)	0.858–0.870	0.877 (0.0001)	0.877–0.878	21.428	<0.001
Model 2	7					374.600	<0.001
Accuracy		0.883 (0.002)	0.882–0.884	0.887 (0.002)	0.886–0.887	52.892	<0.001
TPR		0.826 (0.006)	0.824–0.829	0.843 (0.003)	0.842–0.844	187.103	<0.001
TNR		0.885 (0.002)	0.884–0.886	0.888 (0.002)	0.887–0.889	34.678	<0.001
AUC		0.869 (0.022)	0.860–0.877	0.917 (0.001)	0.916–0.917	140.144	<0.001
Model 3	11					832.822	<0.001
Accuracy		0.868 (0.0003)	0.868–0.869	0.860 (0.003)	0.859–0.861	251.494	<0.001
TPR		0.882 (<0.0001)	0.882–0.882	0.925 (0.004)	0.924–0.927	3184.364	<0.001
TNR		0.868 (0.0003)	0.868–0.868	0.858 (0.003)	0.857–0.859	320.672	<0.001
AUC		0.909 (0.017)	0.902–0.915	0.938 (0.001)	0.938–0.939	95.037	<0.001
Model 4	15					571.875	<0.001
Accuracy		0.868 (0.0004)	0.868–0.869	0.872 (0.004)	0.871–0.874	34.529	<0.001
TPR		0.882 (<0.0001)	0.882–0.882	0.924 (0.006)	0.922–0.927	1,534.352	<0.001
TNR		0.868 (0.0004)	0.868–0.868	0.871 (0.004)	0.869–0.872	15.506	<0.001
AUC		0.924 (0.008)	0.920–0.927	0.951 (0.001)	0.951–0.952	341.328	<0.001
Model 5	17					767.678	<0.001
Accuracy		0.869 (<0.0001)	0.869–0.869	0.875 (0.003)	0.874–0.876	163.709	<0.001
TPR		0.882 (<0.0001)	0.882–0.882	0.924 (0.005)	0.922–0.926	1,970.827	<0.001
TNR		0.868 (<0.0001)	0.868–0.868	0.873 (0.003)	0.872–0.874	101.359	<0.001
AUC		0.923 (0.013)	0.918–0.928	0.953 (0.001)	0.953–0.953	156.308	<0.001

Results of the one-way multivariate analysis of variance (MANOVA) comparing prediction performance between support vector machine and random forest approaches for each model. TPR, true positive rate; TNR, true negative rate; AUC, area under the receiver operating characteristic curve.

### 3.2. Number of prescriptions

**FIGURE 4:**
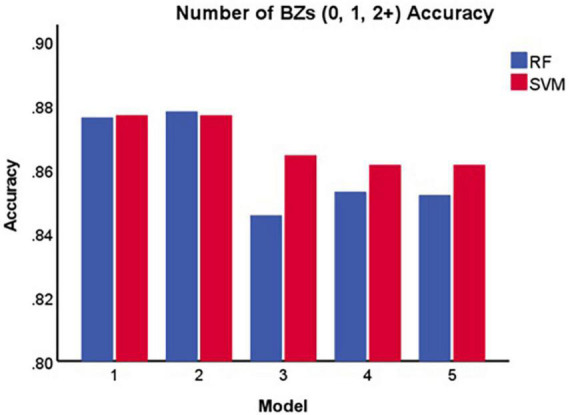
Accuracy of each model in predicting the number of benzodiazepines a patient receives at a given encounter (0, 1, 2+). BZ, benzodiazepine; SVM, support vector machine; RF, random forest.

Table 2 and Figure 4 display the results for the models predicting the number of benzodiazepine prescriptions received at a given encounter (0, 1, 2+). As demonstrated in Figure 4, Model 2 maximized overall accuracy when predicting how many benzodiazepine prescriptions a patient received at an encounter (0, 1, 2+), with the random forest model slightly outperforming the SVM model (Random Forest number of benzodiazepines Model 2 accuracy = 0.878; SVM number of benzodiazepines Model 2 accuracy = 0.877, *F*(1, 28) = 0.808, *p* = 0.372), though this difference was not statistically significant.

**TABLE 2:** Number of benzodiazepine prescriptions (0, 1, 2+) prediction results.

		Support vector machine		Random forest		Comparison	
	Number of features	M (SD)	95% CI	M (SD)	95% CI	*F*	*p*
Model 1	4					2.167	0.085
Accuracy		0.877 (0.006)	0.875–0.879	0.876 (0.007)	0.874–0.879	0.189	0.666
TPR1		0.806 (0.024)	0.800–0.815	0.819 (0.024)	0.810–0.828	4.340	0.042
TPR2		0.081 (0.048)	0.064–0.099	0.051 (0.039)	0.036–0.066	7.223	0.009
TNR		0.880 (0.006)	0.878–0.883	0.879 (0.008)	0.876–0.882	0.346	0.558
Model 2	7					51.346	<0.001
Accuracy		0.877 (0.006)	0.874–0.879	0.878 (0.004)	0.877–0.880	0.808	0.372
TPR1		0.805 (0.024)	0.796–0.814	0.727 (0.020)	0.719–0.734	182.135	<0.000
TPR2		0.084 (0.046)	0.067–0.102	0.220 (0.055)	0.199–0.241	106.706	<0.000
TNR		0.880 (0.007)	0.878–0.883	0.884 (0.004)	0.882–0.885	4.859	0.031
Model 3	11					212.277	<0.001
Accuracy		0.864 (0.006)	0.862–0.867	0.846 (0.004)	0.844–0.847	192.047	<0.001
TPR1		0.851 (0.021)	0.843–859	0.741 (0.021)	0.734–0.749	403.404	<0.001
TPR2		0.071 (0.053)	0.051–0.091	0.307 (0.039)	0.293–0.322	391.945	<0.001
TNR		0.866 (0.007)	0.864–0.869	0.850 (0.005)	0.848–0.851	130.430	<0.001
Model 4	15					44.441	<0.001
Accuracy		0.861 (0.006)	0.859–0.864	0.853 (0.007)	0.850–0.855	25.549	<0.001
TPR1		0.732 (0.019)	0.725–0.739	0.783 (0.013)	0.778–0.788	147.144	<0.001
TPR2		0.371 (0.018)	0.365–0.378	0.380 (0.040)	0.365–0.395	1.248	0.269
TNR		0.866 (0.007)	0.863–0.868	0.856 (0.007)	0.853–0.858	32.896	<0.001
Model 5	17					39.007	<0.001
Accuracy		0.861 (0.006)	0.859–0.863	0.852 (0.007)	0.849–0.854	31.096	<0.001
TPR1		0.732 (0.023)	0.723–0.741	0.789 (0.013)	0.784–0.794	135.405	<0.001
TPR2		0.372 (0.013)	0.367–0.377	0.313 (0.041)	0.298–0.329	54.608	<0.001
TNR		0.866 (0.006)	0.864–0.868	0.855 (0.008)	0.852–0.857	38.426	<0.001

Results of the one-way multivariate analysis of variance (MANOVA) comparing prediction performance between support vector machine and random forest approaches for each model. TPR, true positive rate; TNR, true negative rate; TPR1, true positive rate for 1 benzodiazepine prescription; TPR2, true positive rate for 2 or more benzodiazepine prescriptions.

## 4. Discussion

Benzodiazepines, which are associated with risk of serious adverse effects (5, 6), represent a significant public health burden (2). Research suggests certain clinical (12, 17–19) and demographic factors (9, 10, 12, 14–16) are associated with benzodiazepine use. However, to our knowledge there is no predictive algorithm which exists that can classify patients by whether they are likely to receive a benzodiazepine prescription and the number of benzodiazepine prescriptions they are likely to receive at a given encounter. The present study used SVM and random forest approaches to develop an algorithm to predict whether a patient is likely to receive a benzodiazepine prescription at a given encounter and how many benzodiazepine prescriptions they are likely to receive at a given encounter, which could facilitate efforts to reduce the public health burden of benzodiazepine use and misuse. We took a step-wise approach to developing a prediction model in order to determine which categories of features are needed to predict benzodiazepine prescriptions accurately. Based on this analysis, both SVM and random forest algorithms may accurately classify individuals who receive a benzodiazepine prescription and can separate patients by the number of benzodiazepine prescriptions received, though there are some differences in performance between the approaches. This proof-of-concept study demonstrates the potential of machine learning approaches in identifying individuals to target for prevention efforts to reduce the burden of benzodiazepine use and inadequately treated anxiety and sleep disorders.

For the SVM approach, overall accuracy did not improve beyond Model 1 (i.e., anxiety and sleep disorder diagnoses), while for the random forest approach, Model 2 (i.e., anxiety and sleep disorder diagnoses and demographic characteristics) maximized overall accuracy when predicting whether a patient received a benzodiazepine prescription at a given encounter (yes/no). For both machine learning approaches, Model 2 maximized overall accuracy when predicting the number of benzodiazepine prescriptions received at an encounter (0, 1, 2+). The random forest model slightly outperformed the SVM model for both outcomes of interest. Of note, including additional groups of features beyond anxiety and sleep diagnoses and demographic characteristics did not improve overall accuracy and, in fact, decreased accuracy slightly. This runs counter to prior research suggesting co-prescriptions, comorbid conditions, and insurance status are important predictors of receiving a benzodiazepine prescription (12). It is possible that the predictive value of those factors is better accounted for by sleep and anxiety disorder diagnoses or patients’ demographic characteristics (i.e., race, age, or gender).

It should be noted that although overall accuracy did not improve when more categories of features were added, including co-prescribed medications in both the SVM and the random forest models improved the true positive rate for benzodiazepine prescription receipt, as well as the number of benzodiazepines prescribed, at a given encounter. Furthermore, including other clinical variables (i.e., any mood disorder diagnosis, any psychotic disorder diagnosis, any neurocognitive disorder diagnosis, prescriber specialty) and insurance status (i.e., whether the patient has insurance, type of insurance) improved the true positive rate for two or more benzodiazepine prescriptions. This suggests that decisions about which categories of features to include in a model may be driven by whether the system employing these machine learning methods is motivated primarily by maximizing sensitivity or specificity. For example, given that an intervention to reduce or prevent benzodiazepine prescribing represents a low risk to the patient, some hospital systems may prefer to use a prediction model that maximizes sensitivity, as false positives would not be a major concern. In contrast, if a hospital system is extremely resource-limited, they may prefer to maximize specificity.

In light of interpretation guidelines (38, 39), all of the SVM and random forest models predicting whether a patient received a benzodiazepine prescription at a given encounter (yes/no) tested in the present study demonstrate good to excellent predictive ability. Model 4 yielded the maximum AUC value for the SVM approach, suggesting that including the most relevant diagnoses, demographic characteristics, co-prescribed medications, and other clinical variables maximizes the predictive value for SVM. However, when using the random forest approach, Model 5 yielded the maximum AUC, suggesting insurance status offers additional predictive value. Both of these approaches yielded AUC values in the excellent range, with random forest slightly outperforming SVM. Thus, employing a random forest approach that utilizes all of the categories of features tested in the present study yields the maximum predictive value when evaluated *via* AUC.

One finding of note in the present study is that although overall accuracy is high for both the prediction of whether a patient will receive a benzodiazepine prescription at a given encounter and the number of benzodiazepines received, the true positive rate for identifying patients who received two or more benzodiazepine prescriptions at a given encounter was relatively low for both the SVM and random forest approach. This may be due to the relatively low base rate of patients receiving multiple benzodiazepine prescriptions at an encounter. To account for this obstacle, in the present study the number of positive and negative observations were balanced prior to model training in an attempt to avoid bias. However, despite low base rate questions being widely recognized as a concern in machine learning, the best method for accounting for this imbalance remains an open question (47). Further research is needed to determine how to best predict the likelihood of receiving two or more benzodiazepine prescriptions.

The present proof-of-concept study suggests that we can predict whether an individual is likely to receive a benzodiazepine prescription at a given encounter and how many benzodiazepine prescriptions they are likely to receive based on information from their electronic health record, with good to excellent predictive ability. Future research is needed to determine whether these predictive models could be useful in a clinical context by alerting providers to a patient’s classification and offering suggestions for how to proceed in light of the risks benzodiazepines can pose to patients’ health (5, 6). For example, if the predictive models used in the present study were employed by a hospital system, a message could be triggered by the algorithm in a patient’s chart that informs a provider of the patient’s risk, provides information on first-line treatments for anxiety and sleep conditions, and makes treatment recommendations. This may include suggesting that the provider refer the patient to cognitive behavioral therapy for anxiety or sleep disturbance (48–51), attempt treatment with a selective-serotonin reuptake inhibitor for anxiety (52), and/or offer the patient educational materials on sleep hygiene and coping skills. Further research is needed to determine whether such an intervention reduces the public health burden of benzodiazepine use and inadequately treated anxiety and sleep disorders. Moreover, the same machine learning methods used in the present study could be applied to examine who is likely to convert to higher risk use (e.g., long-term or high-dose use) (5) if provided a benzodiazepine prescription. Similar methods have been successfully applied to the prediction of opioid use disorder onset (53), sustained opioid prescription (34), and opioid overdose (35). In addition, future research should investigate the utility of employing these machine learning models in longitudinal follow-up data to identify patients who, when prescribed a benzodiazepine, are at elevated risk of side effects or other complications. This would allow for prevention efforts to be targeted at patients who are at the greatest risk of suffering the negative consequences of benzodiazepine use.

The present findings should be interpreted in light of the study’s limitations. First, due to the approach used in the present study, we were unable to ascertain which specific features had the best predictive value. Additionally, the models used in the present study did not provide information on the direction of the relationship between features and the likelihood of receiving a benzodiazepine prescription, although the extant literature provides clues. Moreover, we did not control for benzodiazepine prescription history. Therefore, it is possible that a patient had already received a benzodiazepine prescription prior to the encounters examined in the current study or that patients received benzodiazepine prescriptions by other providers not captured in the current dataset. Finally, there may be additional features that were not included in our models but have value in predicting benzodiazepine prescriptions.

Taken together, the present study suggests SVM and random forest predictive models based on anxiety and sleep diagnoses and demographic characteristics can accurately classify individuals who receive a benzodiazepine prescription and can separate patients by the number of benzodiazepine prescriptions received, with random forest slightly outperforming SVM approaches. Moreover, including additional features can improve the AUC. If results are replicated, machine learning approaches may be useful in determining who to target for prevention efforts to reduce the public health burden of benzodiazepine use and misuse.

## Data availability statement

The data analyzed in this study is subject to the following licenses/restrictions: The data warehouse from which the information for this study is derived has been de-identified and date-shifted so that it does not include any protected health information (PHI). Pursuant to 45 CFR 46, use of this database does not meet the definition of human subjects’ research and does not require IRB review. UMMC Faculty, Staffs, and Students can access this dataset. Requests to access these datasets should be directed to Center for Informatics and Analytics, cia@umc.edu.

## Author contributions

KK designed the study in consultation with all co-authors and drafted the manuscript. YZ conducted the machine learning analyses and provided feedback on the methods and results sections. MM, JS, and JR participated in the design of the study. SB provided consultation on clinicians’ benzodiazepine prescription decision-making processes. All authors provided feedback on the manuscript and approved the final manuscript.
