# Supplementary material for: Predicting benzodiazepine prescriptions: A proof-of-concept machine learning approach
Source: Front Psychiatry. 2023 Mar 10;14:1087879. doi: 10.3389/fpsyt.2023.1087879 (PMC10036348; doi:10.3389/fpsyt.2023.1087879)
Supplement: Supplementary file 1 [file Table_1.docx]

Supplementary Material

Predicting Benzodiazepine Prescriptions: A Proof-of-Concept Machine Learning Approach

Kerry L. Kinney^*^, Yufeng Zheng, Matthew C. Morris, Julie A. Schumacher, Saurabh B. Bhardwaj, James K. Rowlett

*** Correspondence:**

Kerry L. Kinney, Ph.D., kkinney@umc.edu

# Supplementary Figures and Tables

**Supplementary Table 1**

|  | **Training Data (January 2020 - December 2021)** | **Test Data (January 2022 - March 2022)** |
| --- | --- | --- |
|  | ***M (SD)*** | ***M (SD)*** |
| Age | 47.29 (21.57) | 53.27 (20.37) |
|  | ***n* (%)** | ***n* (%)** |
| Gender |  |  |
| Female | 24,262 (63.9%) | 9,408 (65.3%) |
| Male | 13,703 (36.1%) | 4,996 (34.7%) |
| Race |  |  |
| Black/African-American | 23,046 (60.7%) | 8.693 (60.4%) |
| White/Caucasian | 14,933 (39.3%) | 5,711 (39.6%) |
| Insurance |  |  |
| Uninsured | 14,729 (38.8%) | 6,106 (42.4%) |
| Medicare/Medicaid | 12,387 (32.6%) | 5,017 (34.8%) |
| Commercial | 10,153 (26.7%) | 3,096 (21.5%) |
| Worker’s Comp | 38 (0.1%) | 11 (0.1%) |
| Other | 672 (1.8%) | 174 (1.2%) |
| Diagnoses |  |  |
| Primary Anxiety Disorder | 3,512 (9.2%) | 1,125 (7.8%) |
| Any Anxiety Disorder | 5,820 (15.3%) | 2,205 (15.3) |
| Primary Sleep Disorder | 916 (2.4%) | 227 (1.6%) |
| Any Sleep Disorder | 2,579 (6.8%) | 848 (5.9%) |
| Any Mood Disorder | 6,116 (16.1%) | 2,417 (16.8%) |
| Any Psychotic Disorder | 434 (1.1%) | 169 (1.2%) |
| Any Neurocognitive Disorder | 2,156 (5.7%) | 787 (5.5%) |
| Other Prescriptions |  |  |
| Opioid | 1,539 (4.1%) | 413 (2.9%) |
| Antidepressant | 7,156 (18.8%) | 2,283 (15.8%) |
| Antipsychotic | 1,974 (5.2%) | 807 (5.6%) |

*Patient-level Sociodemographic Characteristics in the Training and Test Datasets*

**Supplementary Table 2**

*Training Dataset Patient-Level Characteristics by Benzodiazepine Prescription Status*

|  | **No Benzodiazepine Prescription (*n*=36,624)** | **Received Benzodiazepine Prescription (*n*=1,355)** | *p* |
| --- | --- | --- | --- |
|  | ***M (SD)*** | ***M (SD)*** |  |
| Age | 47.12 (21.69) | 51.77 (17.50) | <.001 |
|  | ***n* (%)** | ***n* (%)** |  |
| Gender |  |  | <.001 |
| Female | 23,269 (63.5%) | 993 (73.3%) |  |
| Male | 13,341 (36.4%) | 362 (26.7%) |  |
| Race |  |  | <.001 |
| Black/African-American | 22,586 (61.7%) | 460 (33.9%) |  |
| White/Caucasian | 14,038 (38.3%) | 895 (66.1%) |  |
| Insurance |  |  | <.001 |
| Uninsured | 14,419 (39.4%) | 310 (22.9%) |  |
| Medicare/Medicaid | 11,806 (32.2%) | 581 (42.9%) |  |
| Commercial | 9,708 (26.5%) | 445 (32.8%) |  |
| Worker’s Comp | 37 (0.1%) | 1 (0.1%) |  |
| Other | 654 (1.8%) | 18 (1.3%) |  |
| Diagnoses |  |  |  |
| Primary Anxiety Disorder | 2,683 (7.3%) | 829 (61.2%) | <.001 |
| Any Anxiety Disorder | 4,695 (12.8%) | 1,125 (83.0%) | <.001 |
| Primary Sleep Disorder | 739 (2.0%) | 177 (13.1%) | <.001 |
| Any Sleep Disorder | 2,165 (5.9%) | 414 (30.6%) | <.001 |
| Any Mood Disorder | 5,295 (14.5%) | 821 (60.6%) | <.001 |
| Any Psychotic Disorder | 368 (1.0%) | 66 (4.9%) | <.001 |
| Any Neurocognitive Disorder | 2,047 (5.6%) | 109 (8.0%) | <.001 |
| Other Prescriptions |  |  |  |
| Opioid | 1,419 (3.9%) | 120 (8.9%) | <.001 |
| Antidepressant | 6,142 (16.8%) | 1,014 (74.8%) | <.001 |
| Antipsychotic | 1,564 (4.3%) | 410 (30.3%) | <.001 |

*Note.* Patients were characterized as having received a benzodiazepine prescription if they received at least one benzodiazepine prescription at one or more encounters between January 2020 and December 2021.

**Supplementary Table 3**

*Test Dataset Patient-Level Characteristics by Benzodiazepine Prescription Status*

|  | **No Benzodiazepine Prescription (*n*=13,770)** | **Received Benzodiazepine Prescription (*n*=634)** | *p* |
| --- | --- | --- | --- |
|  | ***M (SD)*** | ***M (SD)*** |  |
| Age | 53.28 (20.53) | 52.92 (16.68) | .600 |
|  | ***n* (%)** | ***n* (%)** |  |
| Gender |  |  | <.001 |
| Female | 8,944 (65.0%) | 464 (73.2%) |  |
| Male | 4,826 (35.0%) | 170 (26.8%) |  |
| Race |  |  | <.001 |
| Black/African-American | 8,521 (61.9%) | 172 (27.1%) |  |
| White/Caucasian | 5,249 (38.1%) | 462 (72.9%) |  |
| Insurance |  |  | <.001 |
| Uninsured | 5,981 (43.4%) | 125 (19.7%) |  |
| Medicare/Medicaid | 4,709 (34.2%) | 308 (48.6%) |  |
| Commercial | 2,907 (21.1%) | 189 (29.8%) |  |
| Worker’s Comp | 11 (0.1%) | 0 (0.0%) |  |
| Other | 162 (1.2%) | 12 (1.9%) |  |
| Diagnoses |  |  |  |
| Primary Anxiety Disorder | 824 (6.0%) | 301 (47.5%) | <.001 |
| Any Anxiety Disorder | 1,687 (12.3%) | 518 (81.7%) | <.001 |
| Primary Sleep Disorder | 180 (1.3%) | 47 (7.4%) | <.001 |
| Any Sleep Disorder | 657 (4.8%) | 191 (30.1%) | <.001 |
| Any Mood Disorder | 2,025 (14.7%) | 392 (61.8%) | <.001 |
| Any Psychotic Disorder | 138 (1.0%) | 31 (4.9%) | <.001 |
| Any Neurocognitive Disorder | 761 (5.5%) | 26 (4.1%) | .073 |
| Other Prescriptions |  |  |  |
| Opioid | 394 (2.9%) | 19 (3.0%) | .821 |
| Antidepressant | 1,836 (13.3%) | 447 (70.5%) | <.001 |
| Antipsychotic | 620 (4.5%) | 187 (29.5%) | <.001 |

*Note.* Patients were characterized as having received a benzodiazepine prescription if they received at least one benzodiazepine prescription at one or more encounters between January 2022 and March 2022.
